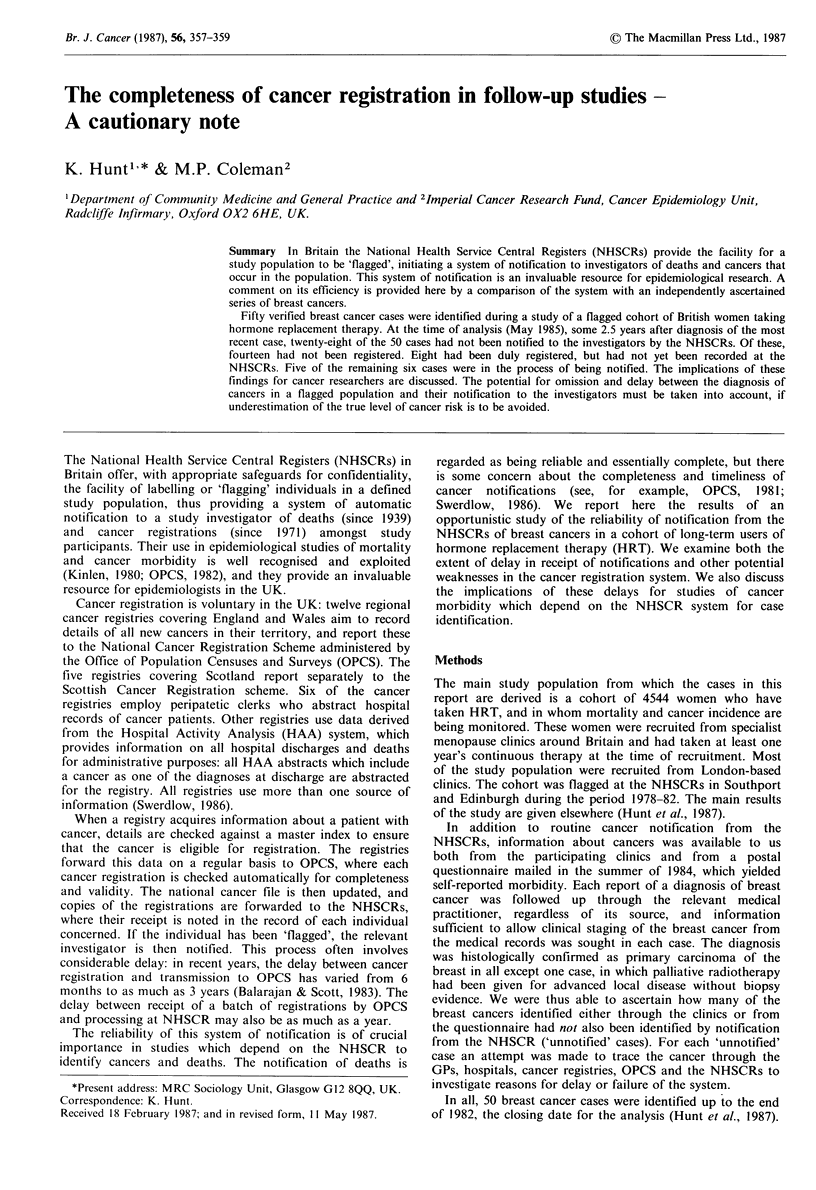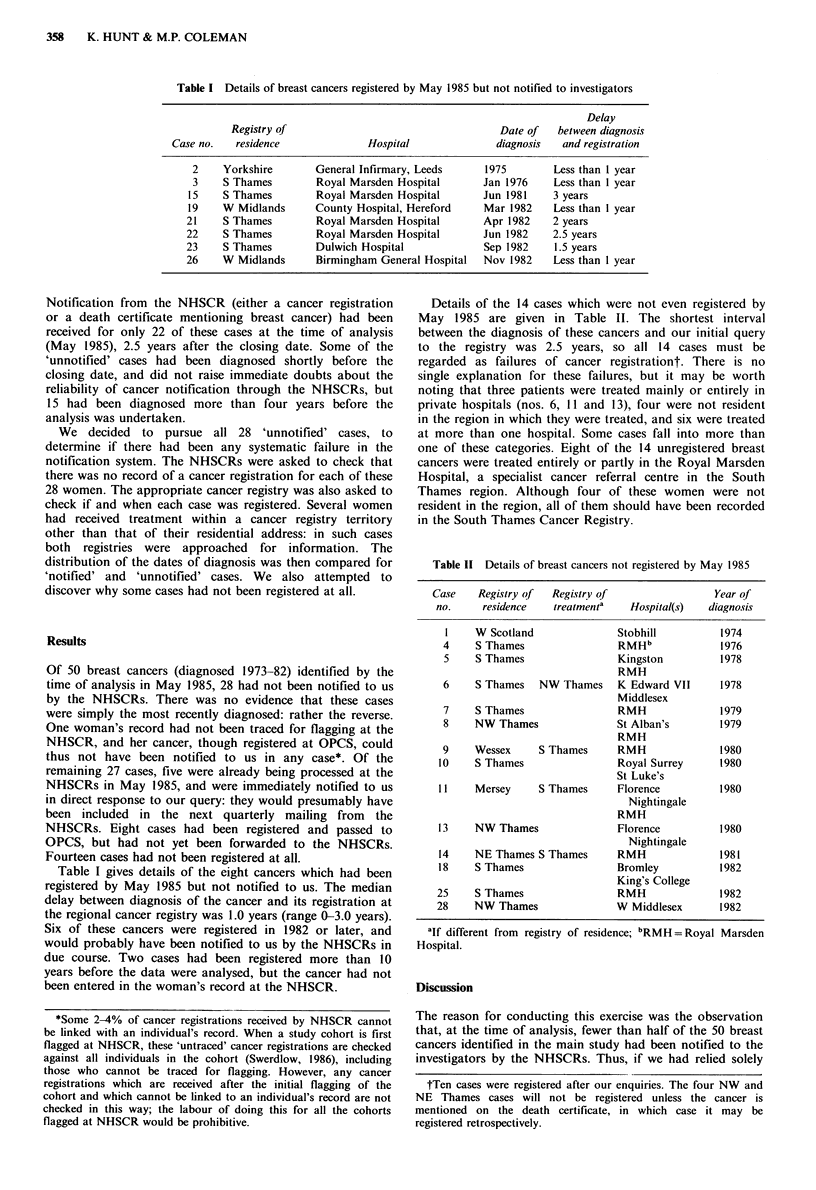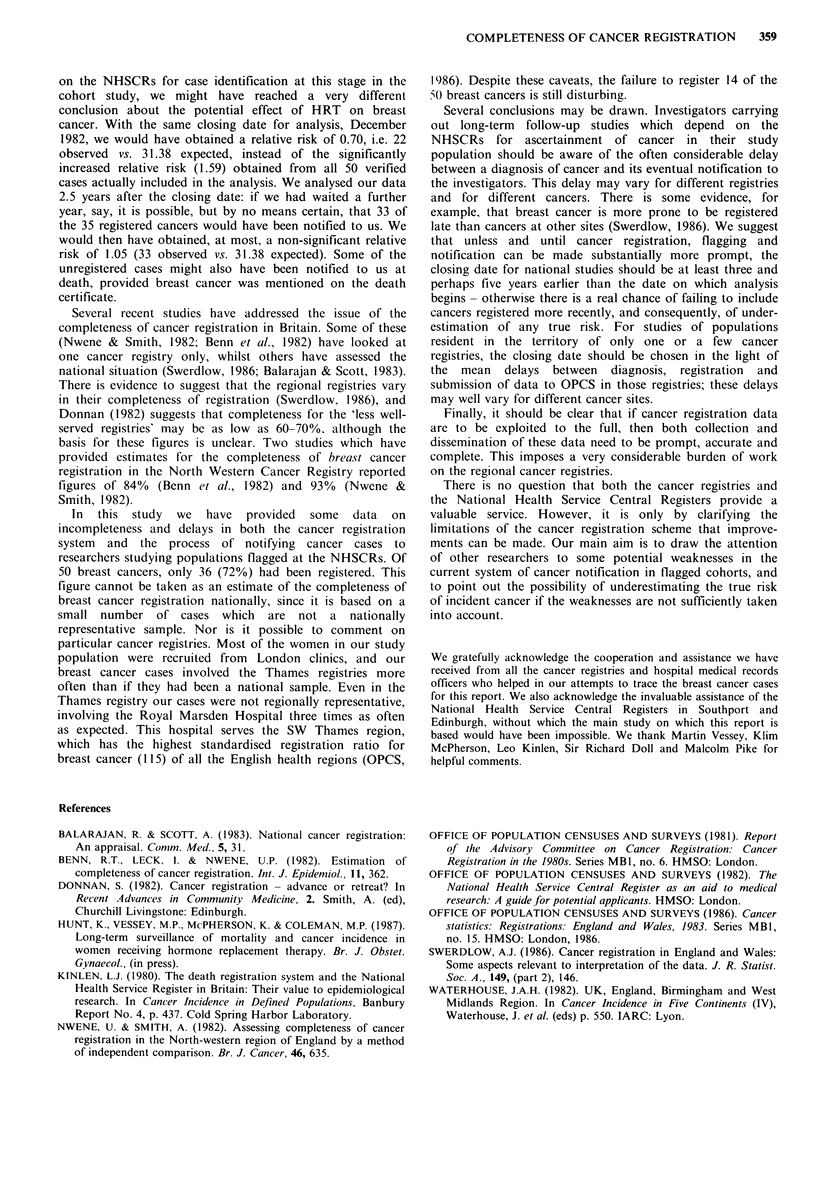# The completeness of cancer registration in follow-up studies--a cautionary note.

**DOI:** 10.1038/bjc.1987.202

**Published:** 1987-09

**Authors:** K. Hunt, M. P. Coleman

**Affiliations:** Department of Community Medicine and General Practice, Radcliffe Infirmary, Oxford, UK.

## Abstract

In Britain the National Health Service Central Registers (NHSCRs) provide the facility for a study population to be 'flagged', initiating a system of notification to investigators of deaths and cancers that occur in the population. This system of notification is an invaluable resource for epidemiological research. A comment on its efficiency is provided here by a comparison of the system with an independently ascertained series of breast cancers. Fifty verified breast cancer cases were identified during a study of a flagged cohort of British women taking hormone replacement therapy. At the time of analysis (May 1985), some 2.5 years after diagnosis of the most recent case, twenty-eight of the 50 cases had not been notified to the investigators by the NHSCRs. Of these, fourteen had not been registered. Eight had been duly registered, but had not yet been recorded at the NHSCRs. Five of the remaining six cases were in the process of being notified. The implications of these findings for cancer researchers are discussed. The potential for omission and delay between the diagnosis of cancers in a flagged population and their notification to the investigators must be taken into account, if underestimation of the true level of cancer risk is to be avoided.


Br. J. Cancer (1987), 56, 357–359 © The Macmillan Press Ltd., 1987

### The completeness of cancer registration in follow-up studies – A cautionary note

K. Hunt[1,]* & M.P. Coleman[2]

[1]*Department of Community Medicine and General Practice and* [2]*Imperial Cancer Research Fund, Cancer Epidemiology Unit, Radcliffe Infirmary, Oxford OX2 6HE, UK.*

**Summary** In Britain the National Health Service Central Registers (NHSCRs) provide the facility for a study population to be 'flagged', initiating a system of notification to investigators of deaths and cancers that occur in the population. This system of notification is an invaluable resource for epidemiological research. A comment on its efficiency is provided here by a comparison of the system with an independently ascertained series of breast cancers.

Fifty verified breast cancer cases were identified during a study of a flagged cohort of British women taking hormone replacement therapy. At the time of analysis (May 1985), some 2.5 years after diagnosis of the most recent case, twenty-eight of the 50 cases had not been notified to the investigators by the NHSCRs. Of these, fourteen had not been registered. Eight had been duly registered, but had not yet been recorded at the NHSCRs. Five of the remaining six cases were in the process of being notified. The implications of these findings for cancer researchers are discussed. The potential for omission and delay between the diagnosis of cancers in a flagged population and their notification to the investigators must be taken into account, if underestimation of the true level of cancer risk is to be avoided.

The National Health Service Central Registers (NHSCRs) in Britain offer, with appropriate safeguards for confidentiality, the facility of labelling or 'flagging' individuals in a defined study population, thus providing a system of automatic notification to a study investigator of deaths (since 1939) and cancer registrations (since 1971) amongst study participants. Their use in epidemiological studies of mortality and cancer morbidity is well recognised and exploited (Kinlen, 1980; OPCS, 1982), and they provide an invaluable resource for epidemiologists in the UK.

Cancer registration is voluntary in the UK: twelve regional cancer registries covering England and Wales aim to record details of all new cancers in their territory, and report these to the National Cancer Registration Scheme administered by the Office of Population Censuses and Surveys (OPCS). The five registries covering Scotland report separately to the Scottish Cancer Registration scheme. Six of the cancer registries employ peripatetic clerks who abstract hospital records of cancer patients. Other registries use data derived from the Hospital Activity Analysis (HAA) system, which provides information on all hospital discharges and deaths for administrative purposes: all HAA abstracts which include a cancer as one of the diagnoses at discharge are abstracted for the registry. All registries use more than one source of information (Swerdlow, 1986).

When a registry acquires information about a patient with cancer, details are checked against a master index to ensure that the cancer is eligible for registration. The registries forward this data on a regular basis to OPCS, where each cancer registration is checked automatically for completeness and validity. The national cancer file is then updated, and copies of the registrations are forwarded to the NHSCRs, where their receipt is noted in the record of each individual concerned. If the individual has been 'flagged', the relevant investigator is then notified. This process often involves considerable delay: in recent years, the delay between cancer registration and transmission to OPCS has varied from 6 months to as much as 3 years (Balarajan & Scott, 1983). The delay between receipt of a batch of registrations by OPCS and processing at NHSCR may also be as much as a year.

The reliability of this system of notification is of crucial importance in studies which depend on the NHSCR to identify cancers and deaths. The notification of deaths is regarded as being reliable and essentially complete, but there is some concern about the completeness and timeliness of cancer notifications (see, for example, OPCS, 1981; Swerdlow, 1986). We report here the results of an opportunistic study of the reliability of notification from the NHSCRs of breast cancers in a cohort of long-term users of hormone replacement therapy (HRT). We examine both the extent of delay in receipt of notifications and other potential weaknesses in the cancer registration system. We also discuss the implications of these delays for studies of cancer morbidity which depend on the NHSCR system for case identification.

### Methods

The main study population from which the cases in this report are derived is a cohort of 4544 women who have taken HRT, and in whom mortality and cancer incidence are being monitored. These women were recruited from specialist menopause clinics around Britain and had taken at least one year's continuous therapy at the time of recruitment. Most of the study population were recruited from London-based clinics. The cohort was flagged at the NHSCRs in Southport and Edinburgh during the period 1978–82. The main results of the study are given elsewhere (Hunt *et al.*, 1987).

In addition to routine cancer notification from the NHSCRs, information about cancers was available to us both from the participating clinics and from a postal questionnaire mailed in the summer of 1984, which yielded self-reported morbidity. Each report of a diagnosis of breast cancer was followed up through the relevant medical practitioner, regardless of its source, and information sufficient to allow clinical staging of the breast cancer from the medical records was sought in each case. The diagnosis was histologically confirmed as primary carcinoma of the breast in all except one case, in which palliative radiotherapy had been given for advanced local disease without biopsy evidence. We were thus able to ascertain how many of the breast cancers identified either through the clinics or from the questionnaire had *not* also been identified by notification from the NHSCR ('unnotified' cases). For each 'unnotified' case an attempt was made to trace the cancer through the GPs, hospitals, cancer registries, OPCS and the NHSCRs to investigate reasons for delay or failure of the system.

In all, 50 breast cancer cases were identified up to the end of 1982, the closing date for the analysis (Hunt *et al.*, 1987).

*Present address: MRC Sociology Unit, Glasgow G12 8QQ, UK.
Correspondence: K. Hunt.
Received 18 February 1987; and in revised form, 11 May 1987.

**Table I** Details of breast cancers registered by May 1985 but not notified to investigators

| Case no. | Registry of residence | Hospital | Date of diagnosis | Delay between diagnosis and registration |
|---|---|---|---|---|
| 2 | Yorkshire | General Infirmary, Leeds | 1975 | Less than 1 year |
| 3 | S Thames | Royal Marsden Hospital | Jan 1976 | Less than 1 year |
| 15 | S Thames | Royal Marsden Hospital | Jun 1981 | 3 years |
| 19 | W Midlands | County Hospital, Hereford | Mar 1982 | Less than 1 year |
| 21 | S Thames | Royal Marsden Hospital | Apr 1982 | 2 years |
| 22 | S Thames | Royal Marsden Hospital | Jun 1982 | 2.5 years |
| 23 | S Thames | Dulwich Hospital | Sep 1982 | 1.5 years |
| 26 | W Midlands | Birmingham General Hospital | Nov 1982 | Less than 1 year |

Notification from the NHSCR (either a cancer registration or a death certificate mentioning breast cancer) had been received for only 22 of these cases at the time of analysis (May 1985), 2.5 years after the closing date. Some of the 'unnotified' cases had been diagnosed shortly before the closing date, and did not raise immediate doubts about the reliability of cancer notification through the NHSCRs, but 15 had been diagnosed more than four years before the analysis was undertaken.

We decided to pursue all 28 'unnotified' cases, to determine if there had been any systematic failure in the notification system. The NHSCRs were asked to check that there was no record of a cancer registration for each of these 28 women. The appropriate cancer registry was also asked to check if and when each case was registered. Several women had received treatment within a cancer registry territory other than that of their residential address: in such cases both registries were approached for information. The distribution of the dates of diagnosis was then compared for 'notified' and 'unnotified' cases. We also attempted to discover why some cases had not been registered at all.

### Results

Of 50 breast cancers (diagnosed 1973–82) identified by the time of analysis in May 1985, 28 had not been notified to us by the NHSCRs. There was no evidence that these cases were simply the most recently diagnosed: rather the reverse. One woman's record had not been traced for flagging at the NHSCR, and her cancer, though registered at OPCS, could thus not have been notified to us in any case*. Of the remaining 27 cases, five were already being processed at the NHSCRs in May 1985, and were immediately notified to us in direct response to our query: they would presumably have been included in the next quarterly mailing from the NHSCRs. Eight cases had been registered and passed to OPCS, but had not yet been forwarded to the NHSCRs. Fourteen cases had not been registered at all.

Table I gives details of the eight cancers which had been registered by May 1985 but not notified to us. The median delay between diagnosis of the cancer and its registration at the regional cancer registry was 1.0 years (range 0–3.0 years). Six of these cancers were registered in 1982 or later, and would probably have been notified to us by the NHSCRs in due course. Two cases had been registered more than 10 years before the data were analysed, but the cancer had not been entered in the woman's record at the NHSCR.

*Some 2–4% of cancer registrations received by NHSCR cannot be linked with an individual's record. When a study cohort is first flagged at NHSCR, these 'untraced' cancer registrations are checked against all individuals in the cohort (Swerdlow, 1986), including those who cannot be traced for flagging. However, any cancer registrations which are received after the initial flagging of the cohort and which cannot be linked to an individual's record are not checked in this way; the labour of doing this for all the cohorts flagged at NHSCR would be prohibitive.

Details of the 14 cases which were not even registered by May 1985 are given in Table II. The shortest interval between the diagnosis of these cancers and our initial query to the registry was 2.5 years, so all 14 cases must be regarded as failures of cancer registration†. There is no single explanation for these failures, but it may be worth noting that three patients were treated mainly or entirely in private hospitals (nos. 6, 11 and 13), four were not resident in the region in which they were treated, and six were treated at more than one hospital. Some cases fall into more than one of these categories. Eight of the 14 unregistered breast cancers were treated entirely or partly in the Royal Marsden Hospital, a specialist cancer referral centre in the South Thames region. Although four of these women were not resident in the region, all of them should have been recorded in the South Thames Cancer Registry.

**Table II** Details of breast cancers not registered by May 1985

| Case no. | Registry of residence | Registry of treatment[a] | Hospital(s) | Year of diagnosis |
|---|---|---|---|---|
| 1 | W Scotland | | Stobhill | 1974 |
| 4 | S Thames | | RMH[b] | 1976 |
| 5 | S Thames | | Kingston<br>RMH | 1978 |
| 6 | S Thames | NW Thames | K Edward VII<br>Middlesex | 1978 |
| 7 | S Thames | | RMH | 1979 |
| 8 | NW Thames | | St Alban's<br>RMH | 1979 |
| 9 | Wessex | S Thames | RMH | 1980 |
| 10 | S Thames | | Royal Surrey<br>St Luke's | 1980 |
| 11 | Mersey | S Thames | Florence Nightingale<br>RMH | 1980 |
| 13 | NW Thames | | Florence Nightingale | 1980 |
| 14 | NE Thames | S Thames | RMH | 1981 |
| 18 | S Thames | | Bromley<br>King's College | 1982 |
| 25 | S Thames | | RMH | 1982 |
| 28 | NW Thames | | W Middlesex | 1982 |

[a]If different from registry of residence; [b]RMH = Royal Marsden Hospital.

### Discussion

The reason for conducting this exercise was the observation that, at the time of analysis, fewer than half of the 50 breast cancers identified in the main study had been notified to the investigators by the NHSCRs. Thus, if we had relied solely

†Ten cases were registered after our enquiries. The four NW and NE Thames cases will not be registered unless the cancer is mentioned on the death certificate, in which case it may be registered retrospectively.

on the NHSCRs for case identification at this stage in the cohort study, we might have reached a very different conclusion about the potential effect of HRT on breast cancer. With the same closing date for analysis, December 1982, we would have obtained a relative risk of 0.70, i.e. 22 observed *vs.* 31.38 expected, instead of the significantly increased relative risk (1.59) obtained from all 50 verified cases actually included in the analysis. We analysed our data 2.5 years after the closing date: if we had waited a further year, say, it is possible, but by no means certain, that 33 of the 35 registered cancers would have been notified to us. We would then have obtained, at most, a non-significant relative risk of 1.05 (33 observed *vs.* 31.38 expected). Some of the unregistered cases might also have been notified to us at death, provided breast cancer was mentioned on the death certificate.

Several recent studies have addressed the issue of the completeness of cancer registration in Britain. Some of these (Nwene & Smith, 1982; Benn *et al.*, 1982) have looked at one cancer registry only, whilst others have assessed the national situation (Swerdlow, 1986; Balarajan & Scott, 1983). There is evidence to suggest that the regional registries vary in their completeness of registration (Swerdlow, 1986), and Donnan (1982) suggests that completeness for the 'less well-served registries' may be as low as 60–70%, although the basis for these figures is unclear. Two studies which have provided estimates for the completeness of *breast* cancer registration in the North Western Cancer Registry reported figures of 84% (Benn *et al.*, 1982) and 93% (Nwene & Smith, 1982).

In this study we have provided some data on incompleteness and delays in both the cancer registration system and the process of notifying cancer cases to researchers studying populations flagged at the NHSCRs. Of 50 breast cancers, only 36 (72%) had been registered. This figure cannot be taken as an estimate of the completeness of breast cancer registration nationally, since it is based on a small number of cases which are not a nationally representative sample. Nor is it possible to comment on particular cancer registries. Most of the women in our study population were recruited from London clinics, and our breast cancer cases involved the Thames registries more often than if they had been a national sample. Even in the Thames registry our cases were not regionally representative, involving the Royal Marsden Hospital three times as often as expected. This hospital serves the SW Thames region, which has the highest standardised registration ratio for breast cancer (115) of all the English health regions (OPCS, 1986). Despite these caveats, the failure to register 14 of the 50 breast cancers is still disturbing.

Several conclusions may be drawn. Investigators carrying out long-term follow-up studies which depend on the NHSCRs for ascertainment of cancer in their study population should be aware of the often considerable delay between a diagnosis of cancer and its eventual notification to the investigators. This delay may vary for different registries and for different cancers. There is some evidence, for example, that breast cancer is more prone to be registered late than cancers at other sites (Swerdlow, 1986). We suggest that unless and until cancer registration, flagging and notification can be made substantially more prompt, the closing date for national studies should be at least three and perhaps five years earlier than the date on which analysis begins – otherwise there is a real chance of failing to include cancers registered more recently, and consequently, of under-estimation of any true risk. For studies of populations resident in the territory of only one or a few cancer registries, the closing date should be chosen in the light of the mean delays between diagnosis, registration and submission of data to OPCS in those registries; these delays may well vary for different cancer sites.

Finally, it should be clear that if cancer registration data are to be exploited to the full, then both collection and dissemination of these data need to be prompt, accurate and complete. This imposes a very considerable burden of work on the regional cancer registries.

There is no question that both the cancer registries and the National Health Service Central Registers provide a valuable service. However, it is only by clarifying the limitations of the cancer registration scheme that improvements can be made. Our main aim is to draw the attention of other researchers to some potential weaknesses in the current system of cancer notification in flagged cohorts, and to point out the possibility of underestimating the true risk of incident cancer if the weaknesses are not sufficiently taken into account.

We gratefully acknowledge the cooperation and assistance we have received from all the cancer registries and hospital medical records officers who helped in our attempts to trace the breast cancer cases for this report. We also acknowledge the invaluable assistance of the National Health Service Central Registers in Southport and Edinburgh, without which the main study on which this report is based would have been impossible. We thank Martin Vessey, Klim McPherson, Leo Kinlen, Sir Richard Doll and Malcolm Pike for helpful comments.

### References

BALARAJAN, R. & SCOTT, A. (1983). National cancer registration: An appraisal. *Comm. Med.*, **5**, 31.

BENN, R.T., LECK, I. & NWENE, U.P. (1982). Estimation of completeness of cancer registration. *Int. J. Epidemiol.*, **11**, 362.

DONNAN, S. (1982). Cancer registration – advance or retreat? In *Recent Advances in Community Medicine*, **2**. Smith, A. (ed), Churchill Livingstone: Edinburgh.

HUNT, K., VESSEY, M.P., McPHERSON, K. & COLEMAN, M.P. (1987). Long-term surveillance of mortality and cancer incidence in women receiving hormone replacement therapy. *Br. J. Obstet. Gynaecol.*, (in press).

KINLEN, L.J. (1980). The death registration system and the National Health Service Register in Britain: Their value to epidemiological research. In *Cancer Incidence in Defined Populations*, Banbury Report No. 4, p. 437. Cold Spring Harbor Laboratory.

NWENE, U. & SMITH, A. (1982). Assessing completeness of cancer registration in the North-western region of England by a method of independent comparison. *Br. J. Cancer*, **46**, 635.

OFFICE OF POPULATION CENSUSES AND SURVEYS (1981). *Report of the Advisory Committee on Cancer Registration: Cancer Registration in the 1980s*. Series MB1, no. 6. HMSO: London.

OFFICE OF POPULATION CENSUSES AND SURVEYS (1982). *The National Health Service Central Register as an aid to medical research: A guide for potential applicants*. HMSO: London.

OFFICE OF POPULATION CENSUSES AND SURVEYS (1986). *Cancer statistics: Registrations: England and Wales, 1983*. Series MB1, no. 15. HMSO: London, 1986.

SWERDLOW, A.J. (1986). Cancer registration in England and Wales: Some aspects relevant to interpretation of the data. *J. R. Statist. Soc. A.*, **149**, (part 2), 146.

WATERHOUSE, J.A.H. (1982). UK, England, Birmingham and West Midlands Region. In *Cancer Incidence in Five Continents* (IV), Waterhouse, J. *et al.* (eds) p. 550. IARC: Lyon.